# An *In-Vitro *Evaluation of Sealing Ability of Real Seal Using Fluid Filtration

**Published:** 2007-04-01

**Authors:** Mina Zarei, Maryam Javidi, Jamileh Ghoddusi, Neda Naghavi, Ehsan Roohani

**Affiliations:** 1 *Department of Endodontics, Member of Dental Research Center, Dental School, Mashad University of Medical Sciences, Mashad, Iran*; 2 *Department of Endodontics, Dental School, Mashad University of Medical Sciences, Mashad, Iran*; 3 *Department of Restorative Dentistry, Dental School, Mashad University of Medical Sciences, Mashad, Iran*

**Keywords:** Fluid Filtration, Real Seal, Sealing Ability, Smear Layer

## Abstract

**INTRODUCTION:** The aim of this study was to compare the sealing ability of Real Seal (RS) and Gutta-percha (GP).

**MATERIALS AND METHODS:** Forty nine extracted human maxillary central incisors were used. The coronal part of each tooth was removed, the root canal was prepared using the crown down technique and apical enlargement to rotary file # 40. The specimens were randomly divided in to 3 groups of 15 each and two control groups of 2 each. Group 1, was obturated with RS and group 2 and 3 were obturated with GP and AH26 sealer by lateral condensation technique. In group 1 and 3 the smear layer was removed by 5mL of 5.25% NaOCl and 3mL of 17% EDTA. Leakage of the obturated roots was measured using the fluid filtration technique. This method was done at 2 min intervals for 8 min. data were analyzed using ANOVA and Tukey tests.

**RESULTS:** Statistical analysis indicated significant differences between groups 2 with 1 and 3. The most leakage value was observed in the group 2.

**CONCLUSION:** Root canal filling with RS or GP in combination with smear layer removal showed better sealing. Therefore the smear layer has more effect one apical leakage than the obturation system.

## INTRODUCTION

For over a century Gutta-percha has been the most commonly used material for obturating the root canal system. Although not the ideal filling material, GP fulfills many of the characteristics that Grossman espoused (1). One of the disadvantages of GP, as a root canal obturation material, is its poor sealing ability; therefore, it must be used with a root canal sealer to provide an effective seal (2). However, when the coronal restoration is defective or absent, contamination with saliva may cause root canal sealer dissolution, thus providing a space for bacterial penetration that may contribute to failure of treatment (3). In addition, dentin removal during root canal treatment has been shown to weaken teeth and make them more susceptible to fracture (4-5).

Obturations with GP do not provide any additional strengthening mechanism for the teeth; therefore, GP-filled teeth may be more prone to fracture than intact teeth (6).

Recently, a thermoplastic synthetic resin polymer, Real Seal, is emerging as a promising root canal obturation material and is gaining popularity among endodontists and general practitioners. Resilon core material is used with the dual cure sealer and self etching primer, this combination purportedly forms a single entity or monoblock in root canal system (7-8). The material has been shown to be non-cytotoxic, biocompatible, and no mutagenic and has been approved for endodontic use by FDA. According to Shipper *et al.* (7), this material has been shown to be more resistant to leakage than GP based obturation systems. The manufacturer also claims that RS has similar handling properties as GP, provides better flexural strength than GP, strengthens the root by more than 20% and can be removed by solvents and heat. Because of its advantages in providing an immediate light-cured seal, RS can potentially offer certain advantages over other root-end filling materials in surgical endodontics (8).

Today, the use of RS as a root-end filling material has not been explored yet. Many *in-vitro* methods have been used to evaluate the sealing ability of root canal filling materials by using dyes, SEM, fluid filtration techniques, electrochemical methods, radioisotopes, and bacteria (9).

This study was designed to compare the sealing properties of Resilon system and GP with AH26 as a sealer. A fluid filtration method was used for quantitative evaluation of leakage in the apical portion of canals.

## MATERIALS AND METHODS

Forty-nine freshly extracted human maxillary incisors with mature apices were selected. Preoperative radiographs were used to ensure that the teeth did not have root caries, fractures, multiple canals, calcifications, radicular resorptions, or excessive curvatures. All teeth were cleaned and stored in normal saline solution until use. The teeth were sectioned at the cemento-enamel junction with a multipurpose bur (Dentsply, Maillefer, Tulsa, OK, USA) and a high speed hand-piece with continuous water spray and the length of roots was adjusted to approximately 16mm. The teeth were randomly divided into three experimental groups of 15 teeth each and two control groups with 2 teeth each. After accessing with a #57 carbide bur (Dentsply International Inc., York, PA, USA), a #15 K-file (Dentsply, Maillefer, Tulsa, Ok, USA) was used to establish apical patency. When the file tip appeared flush with the apical foramen, the length of the file was recorded; the working length was determined to be 1 mm shorter than the measured length. Root canal system was instrumented by a crown-down technique using sequence of 0.06 taper nickel-titanium rotary instruments (Easy race, FKG, Swiss) and hand files (Dentsply, Maillefer, Swiss). All samples were prepared to an ISO size 40. Five milliliters of 5.25% NaOCl was used to irrigate the canals throughout the instrumentation process. In group 1 and 3, the smear layer was removed after instrumentation by using 5 mL of 5.25% NaOCl followed by 3 mL of 17% EDTA (Pulpdent Corp., Watertown, MA). The final rinse was done with 5 mL of physiologic saline (Table 1). In group 2, smear layer was remained; the canals were irrigated only by NaOCl, and physiologic saline as final irrigation (Table 1). The canals were dried with paper points (Dentsply, Maillefer, Swiss).

In the Resilon group (group 1), obturation was done following manufacturer technique pro-tocol. The self-etching primer (Real Seal Primer) was introduced into the canals with sterile paper points to coat the root canal walls and excess primer was removed with sterile paper points. The canals were filled with Resilon and lateral condensation technique.

In two other groups (2 and 3), samples were filled with GP (Dentsply Lexicon, Tulsa, OK, USA) and AH26 (DeTrey, Dentsply, Konstanz, Germany) using the same technique. Each canal orifice was sealed with 3mm of Fuji IX glass Ionomer restorative material (GC America Inc., Alsip, IL, USA).

Root canals of two teeth were prepared, but not obturated, and not sealed prior to leakage testing as positive controls. The remaining two teeth did not instrumented and all surfaces of the root were covered with two layers of Para film (laboratory film, plastic packaging, Chicago) as negative controls.

Specimens were stored at 37oC in 100% humidity for 72 h to complete setting of the sealers and then each teeth was placed in to a device designed for measuring microleakage by fluid transport, first described by Derkson et al. for restorative materials leakage studies and later adapted for endodontic studies by Wu and Wesselink (10).

The root surfaces were covered with two layers of Parafilm (laboratory film, plastic, packaging, Chicago) except the apical 2-3mm. The roots were then connected to a plastic tube with Cyanoacrylate glue (Osaka, Japan) at the apical side and were additionally sealed with Para film. A plastic three valve was connected to another side of plastic tube. A standard glass capillary tube was connected to the three valves. All pipettes, syringes and the plastic tubes at apical sides of the specimens were filled with distilled water. Using a syringe, water was sucked back and an air bubble was created. A pressure of 0.2 atm was applied at the end of the capillary tube to force the water through the voids along the filling, thus displacing the air bubble in the capillary tube. All junctions had been sealed by cianoaccrylate and parafilm.

**Table 1:** Description of experimental groups

**groups**	**Smear layer removal with EDTA 17% and NaOCl 5.25%**	**Filling materials**
1(N=15)	Yes	Real Seal
2(N=15)	No	Gutta-percha /AH26
3(N=15)	Yes	Gutta-percha /AH26

The volume of the fluid transport was measured by observing the movement of the air bubble. The observation was done by a digital camera (Olympus, C 765, Japan) stabilized with distinct distance from micropipette.

The first observation was done 30 sec after pressure use for localization of the bubble and then digital photographs were taken in 2 minutes intervals at 2, 4, 6, and 8 min. Finally, designed software was used for measuring bubble movement and the data were calculated in µL/min/CmH2o and analyzed by ANOVA and Tukey tests.

## RESULTS

The negative controls showed no transportation of the air bubble while, the positive controls showed immediate transportation of the air bubble.

Means and standard deviations of leakage for experimental groups 1, 2, and 3 were 0.0035±.0033, 0.0076±.0030, and 0.0010±.0036 µl/min/cmH2O, respectively.

**Figure 1:**
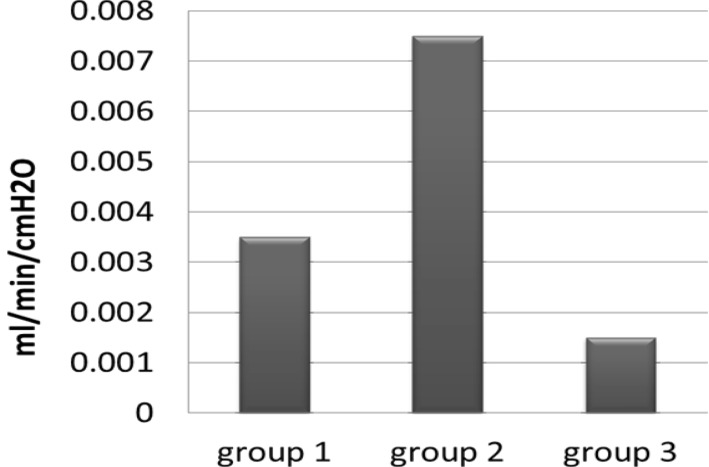
Microleakage in experimental groups

ANOVA test indicated that the difference between three experimental groups was statistically significant (p<0.05). Tukey test was revealed that there were statistically significant differences between group 2 with groups 1 and 3 (Figure 1) (p<0.05).

## DISCUSSION

Several methods have been used to evaluate the sealing ability of root canal filling materials.

The fluid filtration technique is one of the best techniques for quantitative measurement of microleakage of filling materials or apical seal (11). Fluid filtration model has the advantage of not being destroyed so that they can be re-measured.

Among the wide spectrum of commercially available root canal sealers, a new methacrylate resin-based endodontic sealer is designed for bonding simultaneously to intraradicular dentin and filling material, and forming a monoblock (12-13).

Resilon system cause less inflammation in periapical tissues compared to GP/AH26 (8).

In this study, we had 3 groups two of which were filled with GP, same as clinical conditions, with or without smear layer removal, but because of the effect of NaOCl on the bond strength of the primer, in Resilon group we had to use EDTA after NaOCl and before final rinsing with saline, as the manufacturer advocated.

Cobankara *et al.* observed more leakage when the smear layer was not removed (14). The results of the current study were the same and the differences between group 1 with 2 and 2 with 3 were statistically significant and the leakage in group 2 was the most.

Shipper *et al.*, using the microbial leakage evaluation, have shown significantly higher leakage with GP/AH26 than Epiphany/Resilon (7). The present study indicated the same results; however the presence of smear layer had more effect on sealing ability than the obturation system. Biggs *et al.* showed that Resilon/Epiphany was not better than GP/Roth or GP/AH plus at sealing root canals and they concluded that the presence of sealer is necessary for leakage prevention (18).

Numerous investigations have shown that the epoxy resin based sealer has lower leakage than most of other sealers (16-18). Onay *et al.* showed that Resilon sealing ability was not superior to that of GP/AH plus (19), this study also indicated the same results, so more research is needed to determine the sealing ability of Resilon versus other obturation systems in the same experimental condition.

## CONCLUSION

According to the result of present study, when the smear layer is removed the sealing ability of GP and RS is equal. However further study is needed in order to prove this matter.
